# Identification of Loci Associated with Drought Resistance Traits in Heterozygous Autotetraploid Alfalfa (*Medicago sativa* L.) Using Genome-Wide Association Studies with Genotyping by Sequencing

**DOI:** 10.1371/journal.pone.0138931

**Published:** 2015-09-25

**Authors:** Tiejun Zhang, Long-Xi Yu, Ping Zheng, Yajun Li, Martha Rivera, Dorrie Main, Stephanie L. Greene

**Affiliations:** 1 Plant and Germplasm Introduction and Testing Research, United States Department of Agriculture-Agricultural Research Service, Prosser, Washington, United States of America; 2 Department of Horticulture, Washington State University, Pullman, Washington, United States of America; 3 National Center for Genetic Resource Preservation, United States Department of Agriculture-Agricultural Research Service, Fort Collins, Colorado, United States of America; Louisiana State University Agricultural Center, UNITED STATES

## Abstract

Drought resistance is an important breeding target for enhancing alfalfa productivity in arid and semi-arid regions. Identification of genes involved in drought tolerance will facilitate breeding for improving drought resistance and water use efficiency in alfalfa. Our objective was to use a diversity panel of alfalfa accessions comprised of 198 cultivars and landraces to identify genes involved in drought tolerance. The panel was selected from the USDA-ARS National Plant Germplasm System alfalfa collection and genotyped using genotyping by sequencing. A greenhouse procedure was used for phenotyping two important traits associated with drought tolerance: drought resistance index (DRI) and relative leaf water content (RWC). Marker-trait association identified nineteen and fifteen loci associated with DRI and RWC, respectively. Alignments of target sequences flanking to the resistance loci against the reference genome of *M*. *truncatula* revealed multiple chromosomal locations. Markers associated with DRI are located on all chromosomes while markers associated with RWC are located on chromosomes 1, 2, 3, 4, 5, 6 and 7. Co-localizations of significant markers between DRI and RWC were found on chromosomes 3, 5 and 7. Most loci associated with DRI in this work overlap with the reported QTLs associated with biomass under drought in alfalfa. Additional significant markers were targeted to several contigs with unknown chromosomal locations. BLAST search using their flanking sequences revealed homology to several annotated genes with functions in stress tolerance. With further validation, these markers may be used for marker-assisted breeding new alfalfa varieties with drought resistance and enhanced water use efficiency.

## Introduction

Alfalfa (*Medicago sativa L*.) is the most widely grown forage legume crop in the United States and many other countries. Despite its primary value as animal feed, alfalfa also diversifies farming production risks, provides a source of biologically fixed nitrogen for rotational crops, reduces soil erosion, cleans land and water contamination, disrupts pest cycles of annual rotation crops, and promotes soil carbon sequestration. Alfalfa is also a promising crop for use as a bioenergy feedstock [1].

Changing trends to multipurpose uses increases demand for alfalfa. However, the production of alfalfa is challenged by adverse environmental stress factor such as water deficit. Of the world’s usable water resources, 80% is currently consumed by irrigated agriculture. This level of consumption by agriculture is likely not sustainable. Future population growth will require more water for domestic, municipal, industrial, and environmental uses. The most realistic solution is to reallocate a portion of the water currently used by agriculture. Reducing agricultural water use by 10% would increase the amount of water available for other purposes by up to 50% [2]. However, with the world population predicted to reach 9.6 billion by 2050, agriculture is challenged to produce more with less water inputs. This will require substantially more efficient crop production from a smaller irrigation water resource. As a consequence, improving drought resistance and water use efficiency (WUE) is a key for sustainable agriculture. The hotter drier climate predicted with global climate change also makes it imperative that climate resilient crops be developed.

Most alfalfa in the western United States is produced under irrigation. The costs associated with irrigation are significant and are likely to increase as water is diverted to non-agricultural use. Improving WUE of both irrigated and rainfed alfalfa production is imperative.

Drought resistance is an important trait for improving alfalfa productivity under water deficit conditions. Compared to other crop species, little is known about mechanisms by which genetic and physiological factors contribute to drought tolerance in alfalfa. Research on the molecular biology of drought responses in alfalfa was initiated in the 1990s [3–5]. Later on, microarrays were used for analysis of the alfalfa transcriptome to identify genes responsive to dehydration [6]. Most recently, proteomics and metabolite profiling were performed to identify proteins and soluble metabolites that respond to drought in leaves and nodules of alfalfa [7].

Traditional breeding programs and transgenic approaches have been undertaken to improve drought tolerance [8–12]. Modern germplasm has been used for genetic mapping [13,14] and field studies related to forage quality and WUE [13,15–17]. WUE measured by carbon isotope discrimination revealed that *M*. *sativa* ssp. *falcata* had higher WUE compared with other germplasm, although its yield was relatively low [13]. Backcross populations were developed using the *M*. *sativa* ssp. *falcata* variety Wisfal (high WUE) and Chilean germplasm (low WUE) as parents and a genetic map was constructed in these populations [18].

System responses to long-term drought and re-watering of two contrasting alfalfa varieties have been reported [19]. Most recently, quantitative trait loci (QTL) associated with biomass under drought and irrigated conditions have been identified in alfalfa [20].

Gene banks collect thousands of accessions that are potentially useful for plant breeding. In alfalfa this germplasm can include crop wild relatives, wild forms of the domesticated species, primitive landraces and obsolete varieties. Although these materials generally have low agronomic value compared to modern alfalfa cultivars, they may possess useful genes that are not present in the modern crop gene pools. Exploiting these useful genes that were “left behind” is a difficult task because the genes of interest may be tightly linked to unfavorable genes that can be dragged along into breeding populations. Marker assisted selection (MAS) can help speed the process of introgression by helping to identify progeny carrying the traits of interest early in the breeding process. Identifying and exploiting useful alleles in unadapted germplasm with the aim of improving quantitative traits such as drought tolerance and WUE can be particularly challenging, since these traits are most likely under the control of multiple genes and interact with environmental factors. Identification of resistance loci that contribute to variation in such complex traits is a primary challenge in plant breeding and population genetics.

In the present study, we evaluated two important traits associated with drought resistance, drought resistance index (DRI) and relative leaf water content (RWC), in 198 accessions of alfalfa in greenhouse conditions. We used an integrated framework that merges a QTL mapping approach called ‘‘genome-wide association studies (GWAS)” with high-throughput genome sequencing methodologies called “genotyping by sequencing (GBS)” to map traits quickly, efficiently, and in a relatively inexpensive manner. This framework provides a statistical basis for analyzing marker-trait association using linkage disequilibrium. The ultimate objective is to identify molecular markers that can be used for marker-assisted selection for breeding alfalfa cultivars with improved drought resistance and water use efficiency.

## Materials and Methods

### Plant Materials

One hundred and ninety eight alfalfa accessions with potential drought tolerance were selected from the USDA-ARS National Plant Germplasm System (NPGS) alfalfa collection (http://www.ars-grin/npgs.gov). Eighty percent of the accessions had been collected in 1980 during the Canada/United States Germplasm Collecting mission led by Dr. M. Rumbaugh (USDA, ARS, Retired Scientist). The objective of this mission was to collect seed from alfalfa stands that had survived 25 or more years in drought stressed environments. Germplasm was collected in British Columbia, Saskatchewan, Manitoba, Idaho, Montana, Nebraska, New Mexico and North and South Dakota and included varieties such as Grimm and Ladak. The remaining accessions were from different countries, including twelve collected from Afghanistan, two from Bulgaria, China, and Russia, and one from Algeria, India, Lebanon, Saudi Arabia, Spain, Turkey, Oman and Yemen, respectively (Table 1 and S1 Table). Accessions were field tested at the Roza farm at the Irrigated Agriculture Research and Extension Center of Washington State University, Prosser, WA, 2013. Single representative plants were selected and cloned by cutting. The cloned plants were propagated in the USDA-ARS greenhouse, Prosser, WA in 2014. The propagated plants were then transplanted in 6-inches pots containing commercial potting soil (Pro-Mix) and grew in greenhouse at 22°C and 40% relative humidity. In addition to the sun light, extra illumination was added (16 h day/8 h night). The total light density was approximately 200 μmol m^-2^ sec^-1^. The stems were clipped to a length of approximately 5 cm once plants began to flower to generate clones of approximately the same size. The cloned populations were placed in a randomized complete block design with three replications under the same greenhouse conditions.

**Table 1:** Origins and number of accessions of alfalfa germplasm used in the drought tolerance analysis.

International origin	No of accessions	US origin	No of accessions
Afghanistan	12	Idaho	6
Algeria	1	Montana	50
Bulgaria	2	Nebraska	22
Canada	64	North Dakota	18
China	2	South Dakota	11
India	1	New Mexico	1
Lebanon	1		
Oman	1		
Russia	2		
Saudi Arabia	1		
Spain	1		
Turkey	1		
Yemen	1		
Total	90	Total	108

Note: The details of each accession are presented in S1 Table.

### Stress Treatment

All greenhouse plants were cut twice to ensure plant size was uniform. After the second cutting, all pots were irrigated until soil water content reached full soil moisture capacity. Pots were weighed and adjusted to the same weight by adding or reducing wet soil. The treatments were conducted during the early budding period (middle of June, 2014). The control plots were continuously watered and the soil water content remained more than 80%. The drought pots were treated by withholding water. The soil moisture was measured daily using ProCheck with GS3 sensor (Decagon Devices Inc. WA) to estimate the level of drought stress (the average soil water potential was below -0.03 MPa). Stress conditions were maintained until plants showed visible symptoms of wilt for 4 days.

To make the comparisons valid, we kept all plants in the same growing conditions including the temperature, light and soil conditions during the experiment. The soil moisture of each pot was measured daily as described above and used as an indicator for the level of drought stress. In case of potted soil moisture was lower than the average and their variation was higher than 20%, we adjusted to the average level by replenishing water. Therefore, the soil moisture of each pot maintained at the same level and all plants experienced the similar drought stress.

### Measurement of Drought Resistance Index

Biomass yield under drought was measured after the stress treatment. Plants were harvested by cutting at the soil line for measuring biomass yield. Fresh weights of stressed and unstressed plants were used for developing a drought resistance index (DRI, also called drought susceptibility index). DRI is the ratio of yield reduction due to stress in a given genotype as compared to the mean reduction over all genotypes in a given test. DRI is an improved yield measurement over the simple expression of yield under stress as percent of yield under non-stress conditions [21]. The formula of DRI was as follow:

$$\text{DRI}=\frac{Ys}{Yn}/\frac{Ms}{Mn}$$

Where, Ys and Yn are the genotype yields (or biomass) under stress and non-stress, respectively and Ms and Mn are the mean yields (or biomass) over all genotypes in the given test under stress and non-stress conditions, respectively.

### Measurement of Relative Water Content

Relative water content measures the current water content of the sampled leaf tissue relative the maximal water content it can hold at full turgidity [22]. Normal values of RWC range between 98% in fully turgid transpiring leaves to about 30–40% in severely desiccated and dying leaves, depending on plant species. In most crop species the typical leaf RWC at initial wilting is about 60% to 70%, with exceptions.

For each accession, three samples (replications) were taken for each treatment. Each sample represented a different plant. Top-most fully expanded leaves were sampled. Each sample was placed in a pre-weighed airtight vial. Vials were immediately placed in a cooler (around 10–15°C) on ice. Vials were weighed to obtain leaf sample fresh weight (FW), after which the sample was immediately hydrated to full turgidity for 3–4h under normal room light and temperature. Leaflets were hydrated by floating on de-ionized water in a closed petri dish. After hydration the samples were taken out of water and were blotted to remove surface moisture and immediately weighed to obtain fully turgid weight (TW). Samples were then oven dried at 80°C for 24h and weighed (after being cooled in a desiccator) to determine dry weight (DW). The calculation of RWC was done using the following formula:

$$RWC\;\left(\%\right)=\frac{FW-DW}{TW-DW}\times 100$$

Where, FW = fresh weight, TW = turgid weight and DW = dry weight.

### Phenotypic Data Analysis

Phenotypic data collected from three replications of each treatment were first analyzed using Levene’s and T-tests to evaluate the equality of variance and means. Based on the assumption of equal variance, a test for normality was performed on the obtained values for each trait of the association panel with the Kolmogorov-Smirnov tests using the SPSS software (http://www.ibm.com/software/analytics/spss/). Least square means for individuals were calculated by SAS PROC using a mixed model (SAS Institute, NC) and were used for association mapping.

### Genotyping by Sequencing

High molecular weight DNA was extracted from leaves of original plants used to make clones with the Qiagen DNeasy 96 Plant kit, according to the manufacture’s protocol (Qiagen, CA). Genotyping-by-sequencing was carried out as described by Elshire et al. [23]. Briefly, DNA from individuals of the association panel was digested by EcoT221 restriction enzyme, which recognizes a six base-pair sequence (5΄-ATGCA↓T-3΄). Two GBS libraries were prepared by ligating the digested DNA to unique nucleotide adapters (barcodes) followed by PCR amplification. Libraries were placed in two lanes of an Illumina Hi-Seq2000 instrument using 100-base single-end sequencing at Cornell University Sequencing facility (Ithaca, NY). Initial quality check was performed using FastQC (http://www.bioinformatics.babraham.ac.uk/projects/fastqc/). The sequencing reads were deconvoluted and cleaned using the process_rad-tags.pl script in Stacks [24], and 399 million high quality reads retained and aligned to the *M*. *truncatula* reference genome (*Mt* 4.0) (http://phytozome.jgi.doe.gov/jbrowse/index.html?data=genomes%2FMtruncatula&loc) using the Burrow Wheelers Alignment tool (Version 0.5.9) [25] with default alignment parameters. A total of 10% sequence reads was aligned to the *M*. *truncatula* genome. Raw data were submitted to the NCBI Sequence Read Archive with bioproject ID: PRJNA287263 and biosample accession numbers: AMN03779142—SAMN03779330.

### Sequence Variant Detection and Genotype Calling

Alignment data were processed with SAMtools (version 0.1.19) [26] and Picard (version 1.94, http://picard.sourceforge.net/) to mark duplicate reads and estimate the average insert size of the single-end reads. The Genome Analysis Toolkit (GATK) [27] was used for extraction of read-depth information. Read-depth and coverage data were processed with in-house Perl scripts and BEDTools [28].The aligned reads from individuals marked as duplicate were excluded for variant calling. Sequence variants were identified using FreeBayes (Version 0.9.15) [29]. Haplotype-based variants including single and multiple nucleotide polymorphisms (SNPs and MNPs, respectively), allelic series of tri-SNPs and tetra-SNPs, MNPs, and indels with a variable number of nucleotides were identified. A default parameter was used for estimating pairwise diversity. The combined read depth of 10 was used across samples for identifying an alternative allele as a variant, with the minimum mapping and base quality filters of 1 and 3, respectively. The QUAL estimate was used for estimating the phred-scaled probability. Sites with a QUAL value less than 20 were removed. Using these criteria, an error rate of less than 0.01 was obtained.

For genotype calling, “zygosities” of all sequence variants were resolved by allele-specific read-depths for all non-duplicated reads using FreeBayes. The FreeBayes uses Bayes theorem to estimate the probability of a specific set of genotypes within a given number of samples as follow:

$$\text{P}\left(\text{G},\text{S}|\text{R}\right)=\frac{P\left(R|G,S\right)P\left(G\right)P\left(S\right)}{\text{P}\left(\text{R}\right)}$$

Where G = genotype, R = reads, S = sites, P(G) = genotype estimate, P(S) = the mappability of the locus and P(R) = normalizer. This resulted in nulliplex (aaaa), simplex (aaab), duplex (aabb), triplex (abbb) and quadruplex (bbbb) genotype calls relative to the reference sequence for the tetraploid samples. Hence, a variant was called not only if a genotype differs from the reference, but also if a genotype differs from any other genotype. Only variants previously identified by variant calling, and samples with the minimum depth of 10 X at the variant position were used for genotyping.

The heterozygosity of variants was calculated using the R-package “pegas” (http://ape-package.ird.fr/pegas.html) and custom Perl scripts.

### Phylogenic Analyses

The genotype data were used for phylogenic analysis using the cladogram function in TASSEL (http://www.maizegenetics.net/#!tassel/). A neighbor joining tree and default factors were used to analyze genetic relationships among individuals in the association panel and visualized by the online software EvolView (http://www.evolgenius.info/evolview/).

### Genome-Wide Association Analysis

Genotypic data were further filtered using a 5% cutoff value for minor allele frequency. The filtered marker data were transformed into numeric codes (e.g. digitals “0, 1, 2, 3, 4” represent genotypes “aaaa, aaab, aabb, abbb and bbbb”, respectively) before loading to TASSEL. Additional digital codes were assigned to the variants with multiple alleles. A mixed linear model was used for analyzing marker-trait association. The marker data were used to generate a marker similarity matrix (Kinship or K matrix) containing all accessions using TASSEL. TASSEL calculates kinship as the proportion of alleles shared between each pair of lines. Once this matrix is calculated, the numbers are rescaled so that the numbers fall between 0 and 2 (Peter Bradbury, personal communication). The K and Q matrices were used for controlling population structure during association analysis. A false discovery rate (FDR) of 0.05 was used as a threshold for significant association [30].

## Results

### Genetic Diversity of the Panel of Accessions

**Fig 1:**
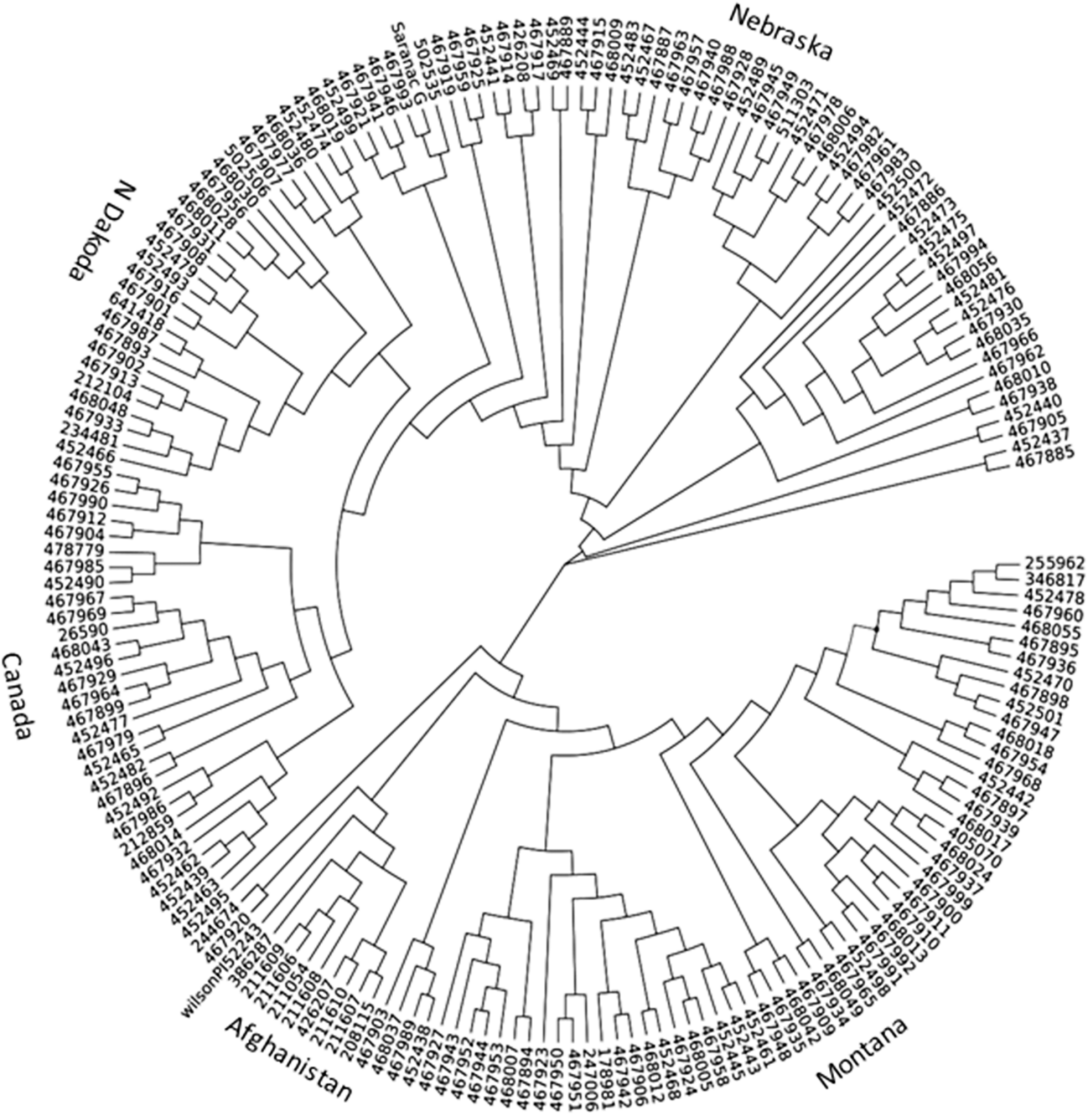
Phylogenic analysis of 198 alfalfa accessions using the marker data generated by GBS and neighbor-joint tree. Their origins are indicated outside of the phylogenic tree.

Among alfalfa accessions used in this study, most were cultivars and were collected from Canada and Northern United States. The rest were from different countries. The genetic backgrounds of germplasm were mostly unknown. To investigate genetic relationships among these accessions, we performed a phylogenic analysis using the marker data generated by GBS and developed a neighbor-joint tree (Fig 1). Generally, accessions with similar origin were clustered together. However, there was no clear bond between clusters except the accessions from Afghanistan. Twelve accessions from Afghanistan and one from Oman were clustered together (Fig 1, lower left). For the most part, accessions from Montana, North Dakota, Nebraska and Canada formed separated clusters. The other accessions were mixed.

### Phenotypic Analysis

**Fig 2:**
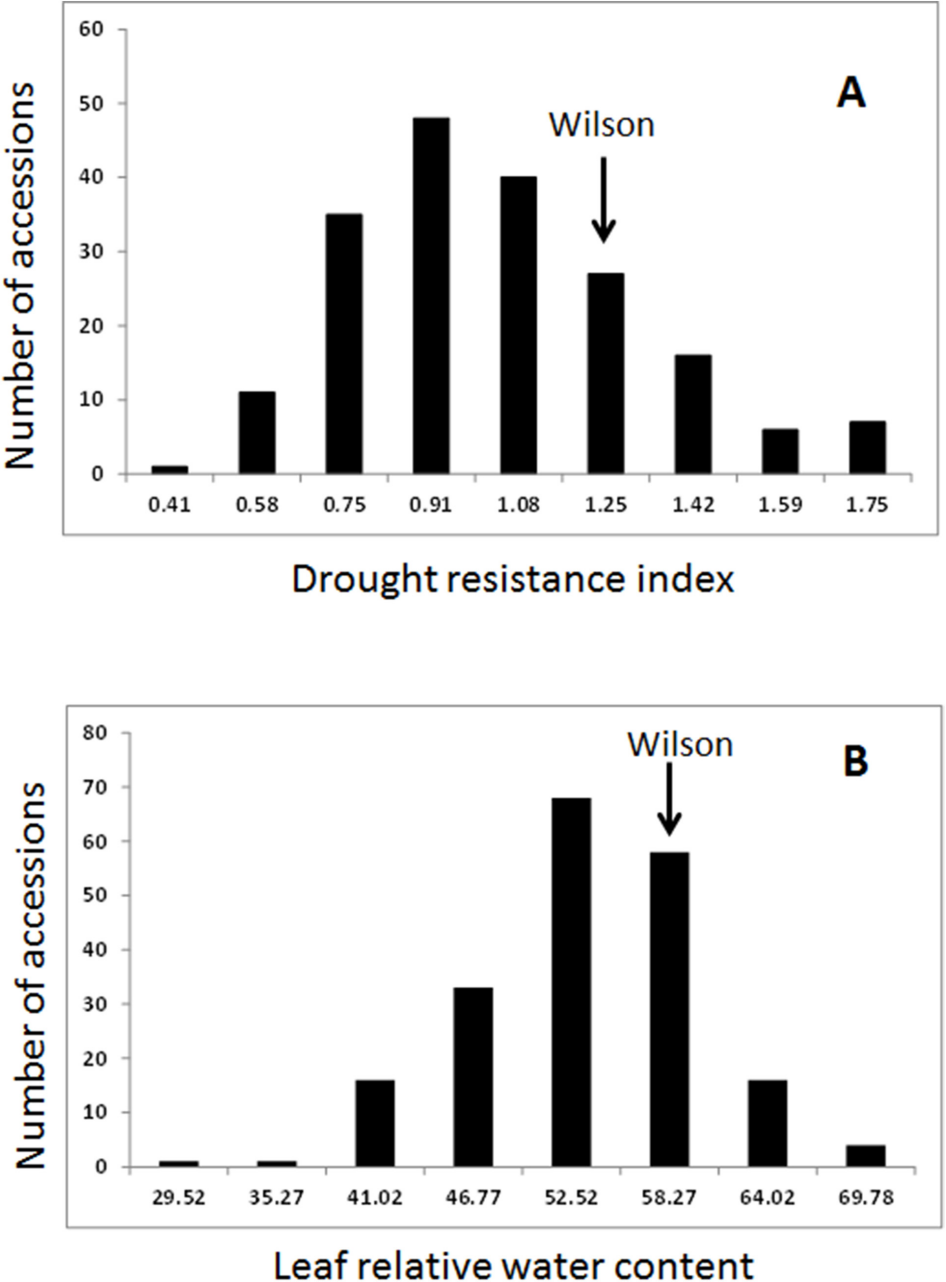
Phenotypic evaluations of drought resistance index (A) and leaf relative water content (RWC). Phenotypic data from three replications were analyzed using a mixed model and least square means were obtained and normal distributions were obtained for both traits (DRI in A and RWC in B). A drought tolerance cultivar, “Wilson” was used as the resistance control for both DRI and RWC experiments.

Similar statistical analyses were used for DRI and RWC, and normal distributions were observed for both DRI and RWC (Fig 2A and 2B).

As fresh weight was used for measuring DRI, It was included as a separate trait in the statistical analysis. Both fresh weight and RWC responded significantly to drought stress (Table 2). The maximum, minimum, and average values of fresh weight and RWC were much lower in drought stressed plants than those of well-watered plants. The average values of fresh weight and RWC were decreased 62.0% and 21.5%, respectively, by drought stress. Significant differences in fresh weight and RWC were found among 198 plants under drought stress, but not in well-watered plants (Table 2). The values of broad-sense heritability (H^2^) of fresh weight and RWC were 0.40 and 0.20, respectively. Similarly, significant difference was observed for DRI. The broad-sense heritability and the genetic variation coefficient for DRI were 0.58 and 18.8%, respectively (Table 2).

**Table 2:** Statistical analyses for phenotyping fresh weight (FW), relative water content (RWC) and drought resistance index (DRI) in 198 alfalfa accessions.

Trait Treatment		Max.	Min.	Ave.	CV.	*F* value	Sig.	H^2^	GCV
FW(g)	Well-watered	36.15	4.78	22.03	22.6%	0.86	0.89	-	-
	Drought	14.30	1.56	8.38	20.5%	1.66	0.001**	0.40	13.0%
RWC (%)	Well-watered	0.83	0.49	0.65	9.07%	0.98	0.56	-	-
	Drought	0.70	0.30	0.51	13.9%	1.24	0.04*	0.20	8.4%
DRI	All	1.58	0.09	1.04	24.2%	2.38	0.001**	0.58	18.8%

Notes: CV, coefficient of variation. H^2^, broad-sense heritability based on accession mean. GCV, genetic variation coefficient. Max = maximum, Min = minimum, Ave = average.

“*” and “**”, significance at *P*≤0.05 and 0.01, respectively.

### Genotype Calling and Allele Frequencies

**Fig 3:**
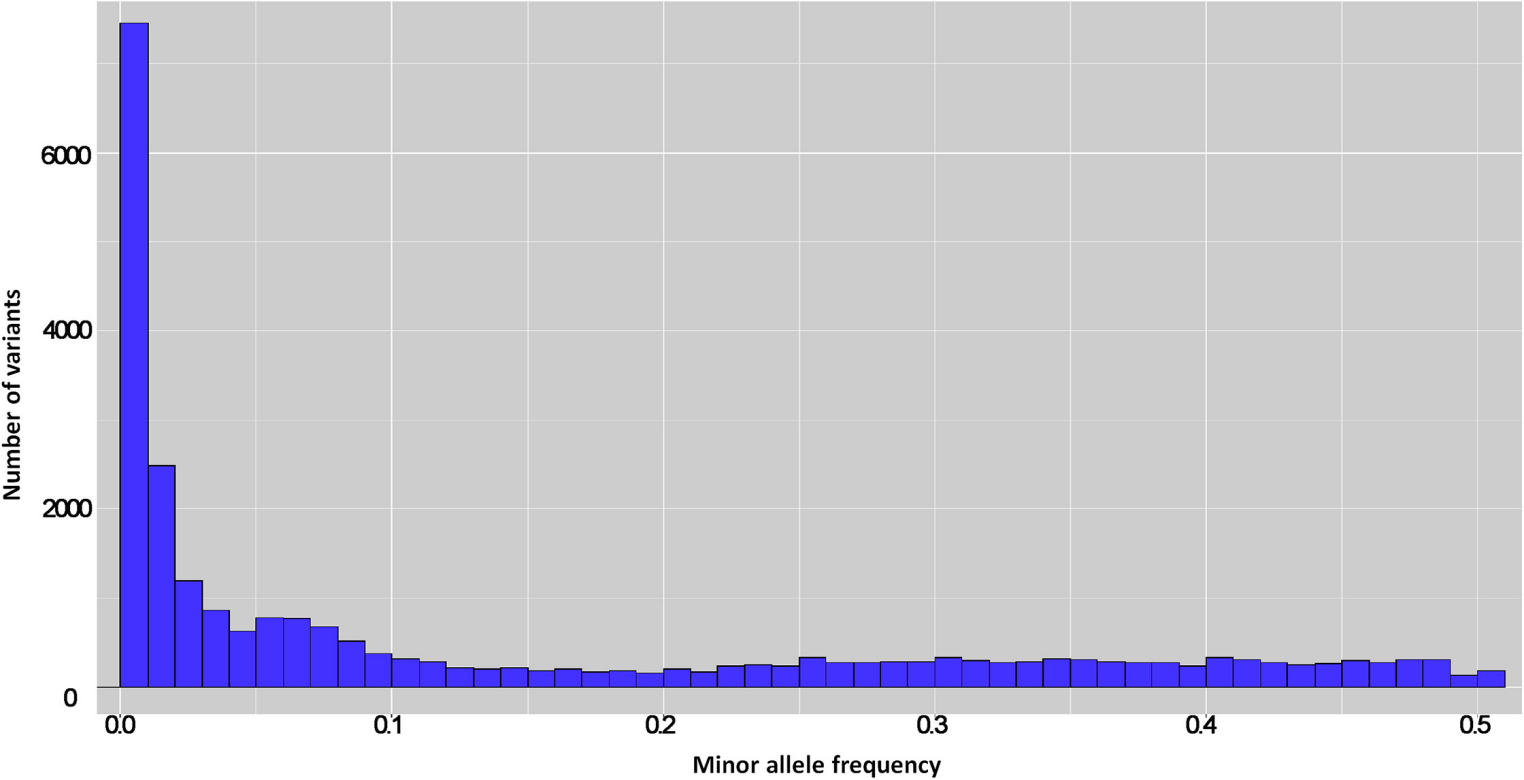
The distribution of minor allele frequency of valid variants calls by genotyping by sequencing.

**Fig 4:**
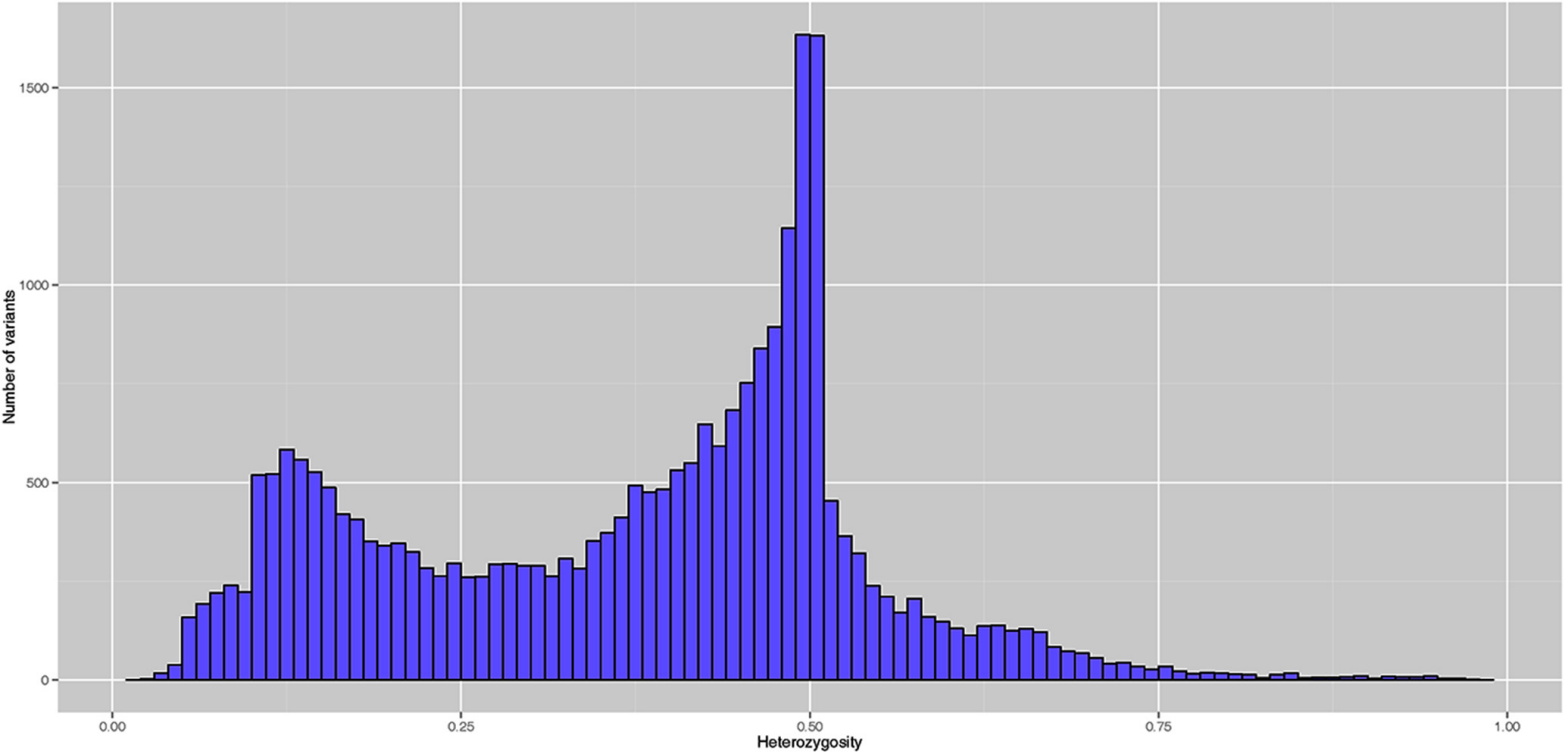
The distribution of heterozygosity of valid variants after genotyping by sequencing pipeline.

Given that alfalfa is an autotetraploid species, each locus may have up to 5 alleles. Commonly used pipelines do not handle tetraploid genotype calling. We therefore used a series of software for sequence alignment and genotype calling in this experiment. A total of 223,362 variants were called with a minimum of 1 alternate count in one individual. Variants with a minimum of 10 read depth and QUAL > 20 at the variant position were used for genotype calling. Of those, 26,163 variants were valid genotype calls after filtering. The average SNP/MNP density in the genome after filtering was 72.1 bp. Population-level allele frequencies were calculated using all valid calls (26,163). The minor allele frequencies (MAF) had a normal distribution as most of variants had lower MAF and the number of variants progressively reduced as MAF increased (Fig 3). The average MAF was 0.14, with 28.5% variants having MAF of 0.01, and 51.2% having MAF of 0.05. The heterozygosity showed a bimodal distribution with two peaks toward 0.1 and 0.5 (Fig 4) which is similar to practical [31] and theoretical observations [32] under assumptions of the neutral theory. The mean heterozygosity of the variants was 0.37. There were 20.9% variants with heterozygosity greater than 0.5 and 4.2% variants with heterozygosity less than 0.1.

### Marker-Trait Association

**Fig 5:**
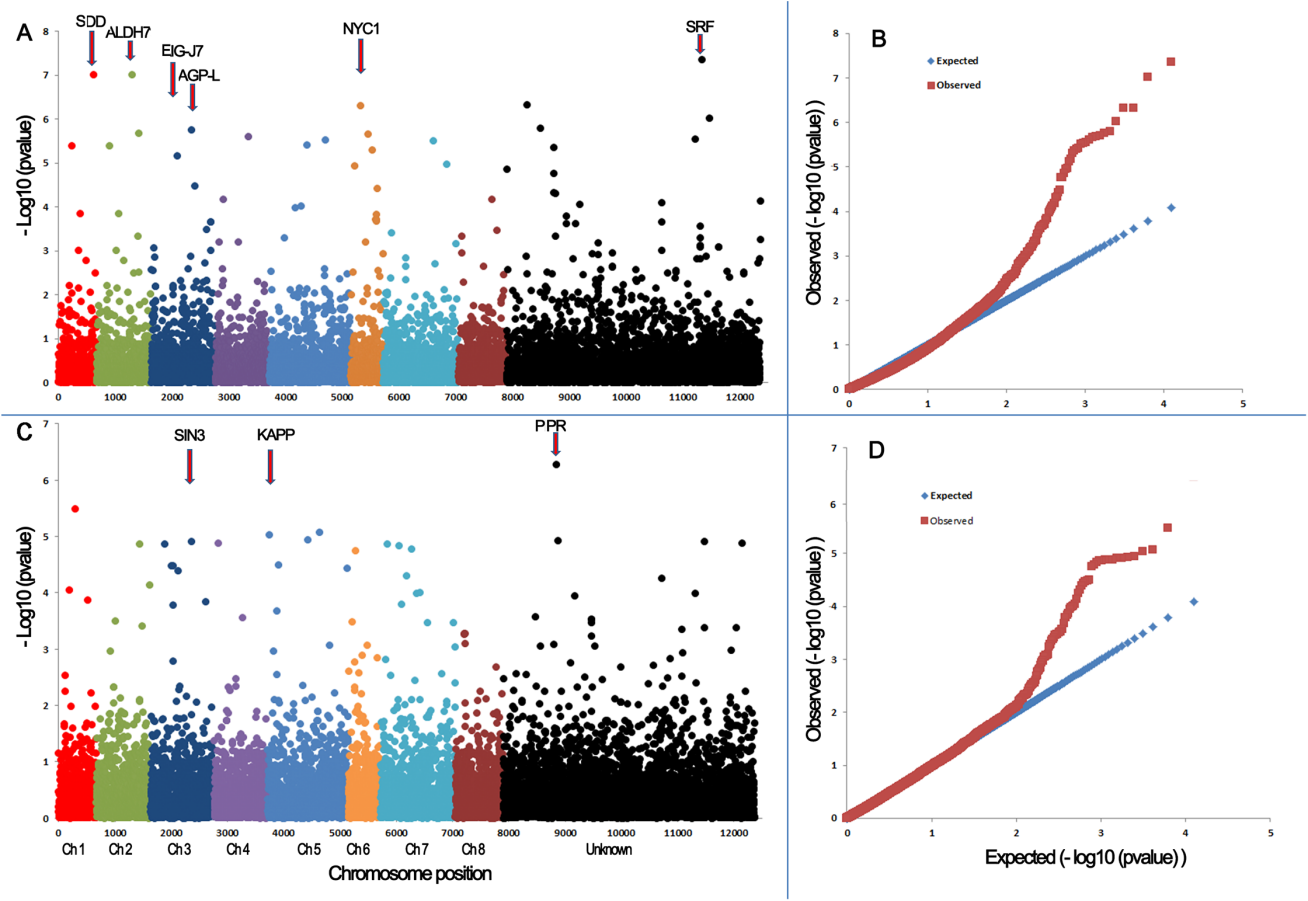
Manhattan and quantile-quantile plots resulting from GWAS for drought resistance index (A, B) and leaf relative water content (C, D) in alfalfa. A false discovery rate of 5% was used for significant cutoff (dot lines). Significant markers (above the cutoff lines) associated with DRI and RWC were listed in Tables 3 and 4, respectively. The chromosome position was based on the reference genome of *M*. *truncatula* sequence (Version 3.5). “U” represents unknown chromosome. The abbreviations at the top of plots are putative candidate genes and the arrows indicate their genetic positions in the reference genome. SDD, subtilisin-like protease; ALDH7A1, Aldehyde dehydrogenase family 7 member A1; EIG-J7, Elicitor inducible protein EIG-J7; AGP-L, Glucose-1-phosphate adenylyltransferase large subunit; NYC1, Oxidoreductase NYC1; SRF, strubbelig-receptor family 3-like protein; SIN3, SIN3 component histone deacetylase complex; KAPP, Kinase-associated protein phosphatase; PPR, pentatricopeptide repeat-containing protein.

Nineteen SNP or MNP markers were significantly associated with drought resistance index and fifteen of them were assigned to 7 chromosomes based on the alignments against the reference genome of *M*. *truncatula* (Fig 5A, Table 3). Marker S1_19681672 was located on chromosome 1 and had 4 variants (CCG, TTG, TCA and TCG). Markers S2_12216981, S2_15380249 and S2_5751832 were located on chromosome 2 and had 2, 4 and 2 variants, respectively. Two significant penta-SNPs, S3_28938985 and S3_42400246 are located on chromosome 3 with 5 and 2 variants, respectively. One tetra-SNP (S4_44343064) was found on chromosome 4 with 2 variants. Two markers, S5_20105344 and S5_7439536 were identified on chromosome 5 with 2 and 3 variants, respectively. Three different types of markers were identified on chromosome 6. Among them, S6_18926195 was a tri-SNP with 3 variants, S6_19756214 was an octa-SNP with 3 variants and S6_21240052 was a tetra-SNP with 3 variants. One tri-SNP (S7_11861405) was found on chromosome 7 with 2 variants. The remaining 4 markers were targeted to different contigs with unknown chromosome location (Table 3, bottom). Among them, contig_66405_1600 and contig_69188_111 were reassigned to chromosome 8 (labeled with *) based on the BLAST search in the *M*. *truncatula* genome (Mt 4.0 v1) using the flanking sequence targets.

**Table 3:** Most significant SNP markers associated with drought resistance index (DRI) in alfalfa.

Trait	Marker	Ref allele	Variant	Chr	p-Value	Effect	Candidate
DRI	1_19681672	TCT	CCG,TTG,TCA,TCG	1	3.94E-06	0.48	SDD
DRI	2_12216981	T	C,G	2	2.03E-06	-0.57	-
DRI	2_15380249	TAAT	CATT,CATC,TCAT,CAAT	2	7.73E-06	-2.61	ALDH7A1
DRI	2_5751832	TAAG	TGAG,TAAT	2	2.05E-06	0.08	-
DRI	contig_113898_4807	TCAAA	CCAAG,TCAAG	2*	1.60E-06	-2.63	-
DRI	3_28938985	CACTA	GACTG,CACTAA,CTCTA,CACCA,CACTG	3	6.71E-06	-2.64	EIG-J7
DRI	3_42400246	CGGAT	TGGAC,CGGAC	3	1.76E-06	-2.62	AGPL
DRI	4_44343064	AGGG	GGGA,GGGG	4	2.44E-06	-2.61	-
DRI	contig_102202_1102	GG	AA,GA	4*	4.74E-07	-2.63	SRF
DRI	5_21015344	G	A,T	5	3.81E-06	0.24	-
DRI	5_7439536	TG	CA,CT,CG	5	2.91E-06	-2.62	-
DRI	6_18926195	GAG	AAC,AAG,GAC	6	2.18E-06	-0.14	-
DRI	6_19756214	TTTGTTGT	TTTGTTGTTGT,TTCATTGT,CTTGTTGT	6	4.84E-07	-2.64	-
DRI	6_21240052	ATTT	GTCT,GTTC,GTTT	6	4.99E-06	-2.64	NYC1
DRI	7_11861405	CAA	CA,CAG	7	3.06E-06	-2.63	-
DRI	contig_66405_1600	TCCT	CCCA,TCTG,TCCA,TCCG	8*	2.81E-06	0.14	-
DRI	contig_69188_111	C	A	8*	4.47E-08	-2.65	-
DRI	contig_72424_3165	CATTTTG	CGTTTA,CGTTTTA,CATTCTA,CATTGTG,CATTTTA	U	9.46E-07	-0.29	-
DRI	contig_122638_1094	TTT	ATA,ATC,CCT,CTT	U	4.39E-06	-2.63	-

Note: Ref allele, allele from the *Medicago truncatula* reference genome sequence assembly (ftp://ftp.ensemblgenomes.org/pub/plants/release-26/fasta/medicago_truncatula/dna/). “Chr”, chromosome assigned based on the marker

position in *Medicago truncatula*. “U”, unknown chromosomal location.

“*”, Unknowns were reassigned to corresponding chromosomes based on the BLAST of the flanking sequence tags against the updated version of *M*. *truncatula* genome (Mt4.0 v1). The full names of putative candidates are listed in Fig 5.

Fifteen markers were significantly associated with RWC and they were assigned to 7 chromosomes based on the alignment against the *M*. *truncatula* genome (Fig 5C and 5D, Table 4). A tetra-SNP, S1_25972745 with 2 variants was assigned to chromosome 1. A tetra-SNP (Contig_94352_776) with 4 variants was also assigned to chromosome 1 based on the BLAST search. A SNP marker, S2_18311315 with 2 variants (T/C or T/G) was assigned to chromosome 2. Two markers S3_28142935 and S3_4235727 were located on chromosome 3. The former was a SNP with one variant (C/T) while the later was a di-SNP with 4 variants. A tetra-SNP (S4_11161035) with 2 variants was identified on chromosome 4. Three markers were identified on chromosome 5, including two tetra-SNPs (S5_28225009 and S5_3579590) with 4 and 3 variants, respectively, and one SNP (S5_396329) with one variant. A tri-SNP (Contig_134721_1800) with 2 variants was aligned to the contig and later assigned to chromosome 6 by homologous search. Three markers were significantly associated with RWC on chromosome 7, including a tetra-SNP (S7_11951427) with 4 variants, a SNP (S7_2982089) with 2 variants, and a di-SNP (S7_31140861) with 3 variants. The remaining 2 markers significantly associated with RWC were homologous to two contigs with unknown chromosome locations. Marker contig_72424_3154 was a tetra-SNP with 4 variants and contig_134721_1180 was tri-SNP with 2 variants.

**Table 4:** Most significant SNP markers associated with leaf relative water content (RWC) in alfalfa.

Trait	Marker	Ref allele	Variant	Chr	p-Value	Effect	Candidate
RWC	1_25872745	ACTA	GCTG,ACTG	1	3.25E-06	0.18	-
RWC	contig_94352_776	CTTT	TTTC,CCTT,ATTT,TTTT	1*	1.30E-05	0.13	-
RWC	2_18311315	T	C,G	2	1.38E-05	0.16	-
RWC	3_28142935	C	T	3	1.34E-05	-0.14	SIN3
RWC	3_4235727	CG	TA,CA,GG,TG	3	1.24E-05	0.22	-
RWC	4_11161035	TGTA	AGTG,AGTA	4	1.32E-05	0.19	-
RWC	5_28225009	AAAT	CAAC,GAAT,AAAA,AAAC	5	8.55E-06	0.03	-
RWC	5_3579590	GTGA	TTGT,GAGA,TTGA	5	9.32E-06	0.16	-
RWC	5_396392	T	C	5	1.15E-05	0.16	KAPP
RWC	contig_131800_1169	ATA	GTG,GTA	6*	5.21E-07	0.14	PRP
RWC	7_11951427	TGTT	CGTA,TTTT,TGCT,TGTA	7	1.37E-05	0.45	-
RWC	7_2982089	C	G,T	7	1.67E-05	0.14	-
RWC	7_31140861	AC	GA,GC,TC	7	1.47E-05	0.17	-
RWC	contig_72424_3154	GCAT	ACAC,ATAT,ACAT	U	1.24E-05	0.46	-
RWC	contig_134721_1800	GGG	TGA,TGG	U	1.18E-05	0.12	-

Note: Ref allele, allele from the *Medicago truncatula* reference genome sequence assembly (ftp://ftp.ensemblgenomes.org/pub/plants/release-26/fasta/medicago_truncatula/dna/) “Chr”, chromosome assigned based on the marker position in *Medicago truncatula*. “U”, unknown chromosomal location.

“*”, Unknowns were reassigned to corresponding chromosomes based on the BLAST of the flanking sequence tags against the updated version of *M*. *truncatula* genome (Mt4.0 v1). The full names of putative candidates are listed in Fig 5.

### BLAST Search for Known Genes Homologs to the Drought Resistance Loci

BLAST search was performed using the flanking sequences of the significant markers identified in the present study against the NCBI nucleotide database (www.ncbi.nlm.nih.gov/). Six homologs were identified for target loci associated with DRI (Table 3, Fig 5A). Among them, a subtilisin-like protease (ADD1) showed high sequence homolog to locus S1_19681672. Aldehyde dehydrogenase family 7 member A1 (ALDH7A1) was homologous to S2_15380249. Elicitor inducible protein EIG-J7 (EIG-J7) was homologous to S3_28938985. Glucose-1-phosphate adenylyltransferase large subunit (AGP-L) was homologous to S3_42400246. Oxidoreductase NYC1 (NYC1) was homologous to S6_21240052 and strubbelig-receptor family 3-like protein (SRF3) was homologous to contig_102202_1102. Three homologs were found for target loci associated with RWC (Table 4, Fig 5B). Among them, SIN3 component histone deacetylase complex; KAPP (SIN3) was homologous to S3_28142935. Kinase-associated protein phosphatase (KAPP) was homologous to S5_396392 and pentatricopeptide repeat-containing protein (PPR) was homologous to contig_131800_1169.

## Discussion

### Markers Associated with Drought Resistance Index

Yield under stress is an important criterion and can be used as a selection index in breeding for water-limited environments. The rate of yield or biomass reduction by stress (e.g. yield under stress as percent of yield under non-stress) is often used as an estimate of resistance in terms of plant production, in addition to absolute yield under stress. This ratio requires tests under both stress and fully irrigated conditions. The drought resistance index is an improvement over the simple expression of yield under stress as the mean yields over all genotypes in the given test under stress and non-stress conditions were used for estimating DRI. In the present study, we identified 19 GBS markers significantly associated with DRI using GWAS. Ray et al. [20] identified 25 SSR markers associated with biomass under water deficit in a biparental alfalfa population and they were located on all chromosomes except chromosome 7. Markers identified by this study were located on all chromosomes (Table 3, Fig 5A). Among them, marker S1_19681672 co-localized with subtilisin-like serine protease (SDD1) whose function involves regulation of stomatal density and distribution in Arabidopsis. It has been suggested that SDD1 acts as a processing protease involved in the mediation of a signal that controls the development of cell lineages that lead to guard cell formation [33]. On chromosome 2, marker S2_15380249 co-localized with aldehyde dehydrogenase family 7 member gene (ALDH7/ATQ1). ALDH7B1 plays a role in regulating turgor pressure in plants. Expression of ALDH7 increased in response to drought stress and is thought to be involved in the regulation of osmotic pressure and protection against oxidative stress in pea [34]. Ectopic expression of the soybean antiquitin-like ALDH7 gene in Arabidopsis and tobacco plants enhanced tolerance to drought, salinity, and oxidative stresses [35].

Of those with unknown chromosome location, marker Contig_102202_1102 was reassigned to chromosome 4 (Table 3) and was found to collocate with strubbelig-receptor family 3 gene (SRF3) encoding a putative leucine-rich repeat receptor-like kinase and playing a role in regulation of leaf size in Arabidopsis [36]. Leaf size is an importance character involved in biomass yield and drought tolerance. It has been reported that cultivars with small leaves reduce water evaporation from plant to atmosphere thus are more tolerant to drought stress [16].

### Markers Associated with Leaf Relative Water Content

Relative water content is probably the most common measure of plant water status in terms of the physiological consequence of cellular water deficit [37]. Schonfeld et al. [38] showed that wheat cultivars having high RWC were more resistant to drought stress. Water potential as an estimate of the energy status of plant water is useful in dealing with water transport in the soil-plant-atmosphere continuum. However, it does not account for osmotic adjustment (OA). OA is a powerful mechanism of conserving cellular hydration under drought stress and RWC can also express the effect of OA in this respect. Two different cultivars with the same leaf water potential can have different leaf RWC, indicating a corresponding difference in leaf hydration, leaf water deficit and physiological water status. Hence RWC is an appropriate estimate of plant water status in terms of cellular hydration under the possible effect of both leaf water potential and OA. In the present study, we identified 15 markers significantly associated with RWC and they were located on 7 chromosomes (Table 4, Fig 5C). Marker associated with RWC on chromosomes 3 and 7 were overlapped with markers associated with DRI. Additional markers for RWC on chromosomes 1, 2, 4, 5, 6 and 8 did not overlap with markers for DRI. Three annotated genes overlapped with markers associated with RWC. Among them, a chromatin-associated protein SIN3-like HDAC complexes (SIN3) located at the S3_28142935 locus interacts with chromatin modeling and the latter in turn plays a role in response to water stress [39]. Another annotated gene, kinase-associated protein phosphatase lined up with S5_396392 has been suggested to regulate adaptation to salt stress in Arabidopsis [40]. A candidate gene encoding pentatricopeptide repeat protein (PPR) lined up with marker Contig_131800_1169. It has been reported that PPR is involved in abiotic stress response in rice [41].

### Comparison of Resistance Loci between Drought Resistance Traits

Although different markers were identified between DRI and RWC, overlaps were appeared for at least three genomic regions. On chromosome 3, marker S3_28938985 identified for DRI closely located to S3_28142935 identified for RWC. Moreover, marker contig_72424_3165 identified for DRI located on chromosome 5 at the same locus of contig_72424_3154 identified for RWC, with only 11 nucleotides apart. Closely located loci were also identified on chromosome 7 between markers S7_11861405 and S7_1191427 associated with DRI and RWC, respectively.

Recently, Ray et al. [20] identified 25 QTLs associated with biomass under drought in two alfalfa mapping populations. Among them, 8 were located on linkage group 1, whereas we identified 2 loci associated with the similar trait DRI. Similarly, in LG2, three loci were identified by Ray et al. [20] and by this study, respectively. Similar chromosomal locations of loci associated with biomass under drought were also identified in other linkage groups by Ray et al [20] and this study except LG7. We identified one locus in LG 7 where none was identified in Ray’s populations [20]. Additionally, in the present analysis, four markers homologous to contigs with unknown chromosomal location were reassigned to LG 2, 4 and 8 based on the BLAST search against the newer version of the *M*. *truncatula* genome sequence (Mt4.0 v1). Among them, two were reassigned to LG 8 where two QTLs were reported by Ray et al. [20].

The ultimate goal of this project is to identify molecular markers for drought stress response and sources of drought tolerance in the primary gene pool to develop superior alfalfa cultivars with drought resistance and enhanced water use efficiency. As a first step, we used GWAS and identified a total of 34 GBS-generated SNP markers that were significantly associated with two important traits related to drought tolerance. Of those, nine markers co-localized with annotated genes in *M*. *truncatula* with potential role in drought response and abiotic stress tolerance. Our next step is to validate these markers with major effects across broader-range of populations in alfalfa using high-throughput platforms such as KASP, Taqman or high resolution melting. After validation, the markers will be used for marker-assisted selection in an alfalfa breeding program to develop superior varieties with drought tolerance and enhanced water use efficiency.

## Supporting Information

S1 Table Detailed information of alfalfa accessions used in drought experiments. (XLSX)
